# Effect of medical insurance policy on child health indicators: an empirical test of difference-in-differences model

**DOI:** 10.3389/fpubh.2025.1559856

**Published:** 2025-07-02

**Authors:** Hui Luo, Jiechuan Fu, Mimi Xiao

**Affiliations:** School of Public Health, Research Center for Medical and Social Development, Chongqing Medical University, Chongqing, China

**Keywords:** medical insurance, integration, child health, physical quality, nutrition, impact lag

## Abstract

**Background:**

The integration policy of urban and rural medical insurance of China is an important policy benefiting the people, aimed at promoting health equity and improving the level of medical security. In the present analysis, we aimed to identify the association between health policy implementation and child health taking China for example.

**Methods:**

Data were drawn from the child sample of China Family Panel Studies (CFPS) 2012–2018, totaling 11,003 items, and the number of illness times, height-for-age *Z*-score, weight-for-age *Z*-score and BMI-for-age *Z*-score were served as health indicators. This paper used the difference-in-differences model to explore the impact of urban and rural medical insurance integration policy on children’s health and the moderating effect model to analyze the mechanism of action.

**Results:**

Our study found that urban–rural medical insurance integration has a positive impact on reducing children’s illness and improving nutritional status, particularly among middle socioeconomic status and rural children. In provinces that implemented the policy in 2017, the realization of the integration policy decreased the number of times of children getting sick (*β* = −0.097, *p* < 0.05), and increased the BMI-for-age *Z*-score (*β* = 0.194, *p* < 0.05). In addition, participation in commercial medical insurance enhanced the positive impact of the integration of urban and rural medical insurance on children’s physical fitness. However, in provinces that implemented the policy in 2018, policy implementation did not change any health indicators. This may suggests a trend where the health promotion effect of the integration policy gradually emerged over time.

**Conclusion:**

It is hoped that this study will provide a policy basis and institutional reference for policy makers to construct and develop the children’s health insurance system.

## 1 Introduction

Universal health coverage (UHC) is a common global goal and a sustainable development necessity. Countries have adopted diverse health insurance policies to achieve it, such as Brazil’s SUS system and India’s PM-JAY scheme (1, 2). China also has actively implemented the urban–rural medical insurance integration policy, establishing the Urban and Rural Residents’ Basic Medical Insurance (URRBMI) to cover all urban and rural residents. This policy aims to eliminate urban–rural health service disparities and promote health equity, which aligns with global reform trends of enhancing fairness and healthcare accessibility (3).

China actively encourages children to legally participate in URRBMI to protect their medical rights, reflecting its attention to children’s health and promoting their healthy growth. Despite China’s progress in child health, as shown by the decline in under – 5 and neonatal mortality rates from 2016 to 2022 (4), inequalities in early childhood development, health – service use, and nutrient intake persist (5–7). As a consequence, it is of great significance to explore the impact of health insurance policies on children’s health, using URRBMI as a starting point for research.

A systematic review of past studies on health insurance policies and insured children shows that most scholars agree that health insurance can enhance the health of specific groups. It is linked to moderate improvements in children’s self-reported health and nutrition (8–10). Health insurance for uninsured children improves their health, cuts out-of-pocket medical costs, and boosts healthcare access (11, 12). However, most studies on the health impacts of urban–rural medical insurance integration focus on adults, while research on its impact on child health is insufficient. Existing related studies mostly concentrate on the pre-integration situation of the New Rural Cooperative Medical Scheme (NRCMS) and the Urban Resident Basic Medical Insurance (URBMI). Some research indicates that NRCMS has the most significant positive impact on infants aged 0–5 (13), correlating with reduced mortality in young children and proven long-term child health benefits, such as preventing underweight conditions (14, 15). Yet, other studies find that NRCMS coverage does not significantly improve rural children’s nutrition (16). URBMI, on the other hand, has increased children’s use of medical services (17). A study using height-for-age Z-scores (HAZ) to measure health shows that participating in URBMI or NRCMS significantly improves child health (18). Another study, using BMI as a measure, points out that public medical insurance participation lowers the probability of obesity in school – aged children and adolescents (19). Moreover, a study using 2018 China Family Panel Studies (CFPS) data, with HAZ, weight-for-age *Z*-scores (WAZ), weight-for-height *Z*-scores (WHZ), and BMI as dependent variables, indicates that URRBMI significantly improves preschool children’s health (20).

In assessing the impact of the urban–rural medical insurance integration policy on child health, there are few existing studies and most of them are cross-sectional analyses. There is currently insufficient evidence for a longitudinal causal relationship between the two. Furthermore, with the policy’s implementation, there’s debate over the sustainability of universal health insurance (21, 22). On one hand, the integration enhances the efficiency and fairness of medical insurance fund use. On the other hand, it causes increased fund expenditure and fiscal pressure. Studying the policy’s impact on child health can inform long – term universal health insurance assessment and adjustment, helping policymakers balance child health protection and medical insurance cost control.

Therefore, this study centers on the impact of urban – rural medical insurance integration on child health indicators. By analyzing the implementation of the URRBMI policy, it can assess the policy’s direct benefits for child health, provide a key basis for improving children’s health protection policies, and offer a valuable reference for other low – and middle – income countries (LMICs) facing similar challenges. This is significant for improving global health governance and promoting health equity in the Sustainable Development Goals.

## 2 Methods

### 2.1 Data sources

China Family Panel Studies (CFPS) were used in our study. CFPS is a national and comprehensive social tracking survey project conducted by Institute of Social Science Survey (ISSS) of Peking University. Its data sample covers 25 provinces, municipalities and autonomous regions and nearly 15,000 households in China. And CFPS questionnaires includes five categories: community questionnaire, family members questionnaire, family questionnaire, children questionnaire and adult questionnaire. This study used data content of family questionnaire, children questionnaire and adult questionnaire.

### 2.2 Sample

In this study, we used data from four waves of CFPS in 2012, 2014, 2016, and 2018. CFPS is a biennial survey with high representativeness and continuity, providing rich and reliable data for studying child-related issues. Our focus is on children under 16, so we drew samples from relevant records in the children’s questionnaire. We only included children covered by URBMI and NRCMS, as studying these groups can directly show the effects of the urban–rural medical insurance integration policy. Before the 2016 “Opinions” were issued, some children in integrated provinces, cities, and districts had different medical insurance policies. To avoid interference, we excluded these samples. This ensures sample consistency in medical insurance background and comparability. In the analysis, we strictly filtered samples. To ensure the accuracy and reliability of the results, we excluded samples missing key information, such as children’s health indicators. We also removed samples lacking complete individual characteristics (such as age, gender, education), family characteristics (such as income, family size), and other related characteristics (such as primary caregivers and their health status). Including complete data ensures accurate model estimation and reliable conclusions. Through these strict selection and exclusion criteria, we built a high-quality, representative, and comparable dataset.

### 2.3 Policy setting

In 2016, the State Council issued the Opinions on Integrating the Basic Medical Insurance System for Urban and Rural Residents, proposing the integration of the Urban Residents’ Basic Medical Insurance (URBMI) and the New Rural Cooperative Medical Scheme (NRCMS), and the implementation of unified medical insurance for rural and urban residents (23). URRBMI is a major initiative in China aimed at achieving health equity and the efficient operation of the insurance system (24). It has brought about three significant changes in the aspects that ordinary families are truly concerned about: population coverage, service items, and cost payment. In terms of population coverage, URBMI initially covered urban non-employed residents, while NRCMS mainly targeted rural residents. This separation caused inequality in medical security between urban and rural residents. With the implementation of the urban–rural medical insurance integration policy, the coverage has been expanded to all urban and rural residents except urban employed ones, eliminating the disparities in health services and security levels between urban and rural areas. Regarding service items, URBMI and NRCMS had different medical insurance catalogs. NRCMS had fewer reimbursable medicines and medical service items, and there were big differences in reimbursement ratios among hospitals of different levels. URRBMI has unified the catalogs of medical insurance medicines and services. Based on the existing catalogs of urban residents’ medical insurance and NRCMS, provinces have formulated unified ones, significantly improving the reimbursement scope and ratio of urban and rural residents’ medical insurance. When it comes to cost payment, NRCMS’ s reimbursement ratio was usually between 50 and 70%, mainly covering hospitalization and serious illness medical expenses, with limited outpatient reimbursement. URBMI’ s reimbursement ratio was slightly higher. Now, the reimbursement ratio of urban and rural residents’ medical insurance is generally between 70 and 90%, with the payment ratio for hospitalization expenses within the policy scope maintained at around 75%. Some areas also offer additional services like chronic disease management, and insured individuals’ out-of-pocket expenses have been significantly reduced.

Since the release of the “Opinions” in 2016, many provincial and municipal governments have clearly integrated URBMI and NRCMS. The unified operation of URRBMI was announced successively in 2017 (10 provinces and cities), 2018 (7 provinces and cities) and 2020 (5 provinces and cities), as shown in Table 1. By the end of 2020, the basic medical insurance system for urban and rural residents in 31 provinces, municipalities directly under the central government and autonomous regions had been in steady operation. We analyzed the differences between the samples of insured children in the 2017 wave and the 2018 wave. We did not choose the 2020 wave, because all provinces and cities in the 2020 wave had been subjected to policy intervention, and there was no suitable control sample. Therefore, the treatment group comprised regions affected by the 2017 or 2018 policies and facing new policy environments after implementation. The control group included regions unaffected by these policies and experiencing no relevant policy changes during the same period. This study only selected the policy waves in 2017 and 2018 for the treatment group, for the following reasons. First, after the official issuance of the “Opinion” in 2016, many provinces and cities actively responded and concentrated on advancing and implementing relevant policies in 2017 and 2018. Second, the earlier policies, due to their long-term implementation and the interweaving of various complex factors during this period, may have their impacts covered or distorted by other factors, making it hard to accurately distinguish their independent effects. Finally, as the later-stage policies had achieved extensive coverage, it seemed challenging to find a control group that is highly comparable to the treatment group in terms of background conditions, etc.

**Table 1:** Implementation time of URRBMI in 31 provinces and cities nationwide.

Years	Provinces or cities	Amounts
2010	Tianjin	1
2012	Guangdong, Chongqing, Ningxia	3
2013	Qinghai	1
2014	Zhejiang, Shandong	2
2016	Shanghai, Fujian	2
2017	Hebei, Jiangxi, Hubei, Hunan, Guangxi, Inner Mongolia, Yunnan, Hainan, Sichuan, Henan	10
2018	Beijing, Xinjiang, Shanxi, Heilongjiang, Jiangsu, Gansu, Anhui	7
2020	Liaoning, Guizhou, Jilin, Shaanxi, Xizang	5

### 2.4 Design of variables

#### 2.4.1 Explanatory variables

The explanatory variable is the product of the policy grouping variable and the policy staging variable. The policy grouping variable takes a value of 1 for the intervention group and 0 for the control group, while the policy staging variable is 0 before the intervention point and 1 afterward. In this study, 2017 and 2018 were designated as policy intervention points, and the available data included the years 2012, 2014, 2016, and 2018. Thus, the health effect on children in provinces that carried out the integration policies in 2017 was the effect after 1 year. Since the policy implementation in the provinces for 2018 occurred at the beginning of the year, and the data survey for 2018 was conducted in July, the impact of the provinces that adopted the integration policy in 2018 is represented as occurring at least 6 months later.

#### 2.4.2 Outcome variables

Outcome variables measuring child health used the number of illness times in the past month, height-for-age Z-score (HAZ), weight-for-age *Z*-score (WAZ), and BMI-for-age *Z*-score (BMIZ). The latter three as measures of malnutrition in children, were calculated according to the WHO standards of children’s growth and development. Individuals with HAZ > 6 or HAZ ≤ −6, WAZ > 5 or WAZ ≤ −6, and BMIZ > 5 or BMIZ ≤ −5 were treated as outliers to remove.

#### 2.4.3 Covariate

Based on previous studies (25–27) our analysis incorporated a comprehensive set of control variables covering personal, family socioeconomic, and health environment characteristics. Personal characteristics included gender (male, female), age groups (0–5, 6–10, 11–15 years old), school attendance (yes, no), left-behind status (yes, no), and birthweight (normal birth weight, low birth weight, macrosomia). Family socioeconomic characteristics covered household registration status (non-agricultural, agricultural), household per capita net income quantiles (bottom 25%, middle and lower 25%, upper 25%, top 25%), and family size (2–7 persons, 8–14 persons). Parents’ education level (primary, secondary, higher education) was also considered. Health environment characteristics included primary caregiver (parental care, grandparental care, other), parents’ health status (unhealthy, healthy), and caregiver’s response to child illness (scientific medical treatment, self-treatment).

Moreover, in the heterogeneity analysis, regions were categorized as eastern, central, or western based on the geographical locations of the sampled provinces. When constructing the socioeconomic status (SES) indicator, we measured it from the two aspects of parents’ education and family income in accordance with relevant literature (28–30). In dealing with the parents’ education variable, we divided it into three levels—primary education, secondary education, and higher education—based on educational stages and assigned them the values of 1, 2, and 3, respectively, to quantify the differences in SES represented by different educational stages. For the family income variable, we cleverly utilized the family per capita income percentile variable in the original data for analysis. This variable was calculated by dividing the family’s total income by the number of family members and was then clearly divided into four levels, namely the lowest family per capita income, relatively low family per capita income, relatively high family per capita income, and the highest family per capita income, to which the values of 1, 2, 3, and 4 were assigned respectively, thereby providing a clear quantitative standard for family income levels. After quantifying and assigning values to these two key variables of parents’ education and family income, we added the scores of these two dimensions to obtain a comprehensive score. Subsequently, based on the comprehensive score, we used the tertile method (dividing the data into three equal parts) to divide it into three distinct levels, thereby constructing an SES indicator covering the three levels of low, medium, and high. This laid a solid foundation for the subsequent in-depth study of the characteristics of different SES groups.

### 2.5 Statistical analysis

The mean and standard deviation were used to describe the quantitative variables HAZ, WAZ, BMIZ and the number of times of sick. Categorical variables were described using frequency and composition ratio. Chi-square test and Kruskal–Wallis rank sum test were used to compare the differences between the intervention and control groups.

The difference-in-differences (DID) method, a robust quasi-experimental approach, enables precise estimation of policy effects by contrasting the changes in outcomes between treatment and control groups pre- and post-policy implementation. This method effectively isolates the policy’s true impact, minimizing confounding factors. The URRBMI offers more benefits than its predecessors, such as premium subsidies and increased reimbursement ratios. In the DID model, the changes in these policy features are incorporated into the policy period dummy variable. By comparing the differences between the intervention group (where the policy is implemented) and the control group (where the policy is not implemented) before and after the policy implementation, the impact of the policy can be accurately assessed. So for studying urban–rural medical insurance integration’s effect on children’s health, DID is invaluable. It was employed here to analyze the integration of the two insurances, establish the causal link between URRBMI and children’s health, and assess the *β* coefficient and 95% confidence interval (95% CI), with statistical significance set at *p* < 0.05. All analyses were conducted using STATA version 17.

Specific model (Equation 1) expression is set as follows:


(1) $$\text{Healt}h_{\mathrm{itp}}=\alpha +\text{δIntegration}+\mu_{i}+\lambda_{t}+\eta X_{\mathrm{itp}}+\varepsilon_{\mathrm{itp}}$$

*Health_itp_* is the explained variable, that is, child health, *i* is the *i* th individual, *p* is the province of the individual, *t* is the year, *X_itp_* is the controlled variable, *μ_i_* is the individual fixed effect, *λ_t_* is the time-fixed effect, ɛ_itp_ is the random error term. The core explanatory variable *Integration* is the product of *Treat_itp_* and *Time_ip_*. *Treat_itp_* indicates whether an individual is subjected to a policy intervention, 1 yes, 0 no. *Time_ip_* means that individuals are observed before or after intervention, 1 after intervention, and 0 before intervention. The interaction term coefficient *δ* reflects the actual effect of the medical insurance integration policy.

To ensure regional comparability in the analysis, we accounted for the varying policy implementation timelines across regions. Macroscopically, we categorized regions into East, Central, and West, following China’s conventional classification. This approach acknowledges disparities in economic development, resource endowment, industrial structure, and policy support among regions. Microscopically, we delved into the family level, strictly controlling for family SES. By doing so, we aimed to mitigate the impact of SES differences on the analysis, ensuring families from diverse regions are assessed under comparable conditions. This method enhanced the study’s accuracy and credibility.

Moreover, we further explored the mechanism by which the integration of urban and rural medical insurance policies affects children’s health indicators. We used whether or not to participate in Commercial medical insurance (CMI) as a moderating variable to conduct an in-depth analysis of the potential impact of participating in CMI on the relationship between urban and rural medical insurance integration policies and children’s health.

Specific model (Equation 2) expression is set as follows:


(2) $$\begin{array}{l} \text{Healt}h_{\mathrm{itp}}=\alpha +\text{δIntegration}+\text{βCMI}+\text{γIntegration}*\mathrm{CMI}+\\ \mu_{i}+\lambda_{t}+\eta X_{\mathrm{itp}}+\varepsilon_{\mathrm{itp}} \end{array}$$

Among them, *β* is the coefficient of the dummy variable *CMI* for participating in Commercial medical insurance, *γ* represents the interaction term coefficient, which is the product of *Integration* and *CMI*. The sign (positive or negative) and magnitude (absolute value) of this coefficient reflect the degree to which the moderating variable enhances or suppresses the main effect. The rest of the components are consistent with the setup of model (Equation 1).

### 2.6 Ethics approval

Data from our study are from China Family Panel Studies (CFPS), which was approved by the Biomedical Ethics Committee of Peking University (IRB00001052-14010) and obtained Informed consent by respondents. This article does not include any research on biological samples of human participants. Before the analysis, all personal information was anonymous and unidentified.

## 3 Results

### 3.1 Descriptive results

Table 2 presents descriptive results for overall and grouped child characteristics. This study covered 11,003 samples, with the 6–10-year-old group being the largest. Of these children, 53.58% (5,895) were boys and 46.42% (5,108) were girls. 16.67% were from urban areas and 83.33% from rural areas. 39.94% were left-behind children and 60.06% were not. Most were in school (77.62%), mainly cared for by their father or mother (45.48%), had healthy mothers (91.21%), healthy fathers (93.35%), had normal birth weight (78.69%), and their guardians chose scientific medical care when they were ill (61.10%). The average values of illness episodes, HAZ, WAZ, and BMIZ were 0.40 ± 0.81, −0.53 ± 1.95, 0.07 ± 1.33, and 0.33 ± 1.68, respectively. Table 2 also presents the test results of the differences in characteristics between the intervention and control groups. The results of *t*-tests, Kruskal–Wallis rank – sum tests, or chi-square tests show that, except for gender, birth weight, and parents’ health, there were significant differences between the two groups in personal, socioeconomic, and family health environment characteristics (*p* < 0.05). For personal characteristics, the intervention group had more rural household registration, fewer children in school, and more left-behind children than the control group. In terms of family socioeconomic characteristics, the intervention group had a slightly larger household size, a higher proportion of high-income families, and a higher proportion of parents with primary or secondary education. In health environment characteristics, the intervention group had a higher proportion of being cared for by grandparents and a higher proportion of choosing scientific medical treatment methods than the control group.

**Table 2:** Demographic characteristics, family characteristics, and health status of insured children, by policy implemented or not*.

Variables	Description	Total (*N* = 11,003)	Control group (*N* = 2035)	Intervention group (*N* = 8,968)	*P*-value
Gender	Female	5,108 (46.42)	931 (45.75)	4,177 (46.58)	
	Male	5,895 (53.58)	1,104 (54.25)	4,791 (53.42)	0.49
Age group	0–5	3,422 (31.10)	582 (28.6)	2,840 (31.67)	
	6–10	4,276 (38.86)	744 (36.56)	3,532 (39.38)	
	11–15	3,305 (30.04)	709 (34.84)	2,596 (28.95)	<0.01
Education	Not yet at school	2,462 (22.38)	416 (20.44)	2046 (22.81)	
	At school	8,541 (77.62)	1,619 (79.56)	6,922 (77.19)	0.02
Hukou	Non-agricultural	1834 (16.67)	434 (21.33)	1,400 (15.61)	
	Agricultural	9,169 (83.33)	1,601 (78.67)	7,568 (84.39)	<0.01
Birth weight	Normal birth weight	8,658 (78.69)	1,612 (79.21)	7,046 (78.57)	
	Low birth weight	525 (4.77)	82 (4.03)	443 (4.94)	
	Macrosomia	1820 (16.54)	341 (16.76)	1,479 (16.49)	0.22
Left-behind children	No	6,608 (60.06)	1,360 (66.83)	5,248 (58.52)	
	Yes	4,395 (39.94)	675 (33.17)	3,720 (41.48)	<0.01
Caregiver	Parental care	5,004 (45.48)	940 (46.19)	4,064 (45.32)	
	Grandparental care	2,615 (23.77)	366 (17.99)	2,249 (25.08)	
	Other	3,384 (30.76)	729 (35.82)	2,655 (29.61)	<0.01
Father’s health	Unhealthy	732 (6.65)	128 (6.29)	604 (6.74)	
	Healthy	10,271 (93.35)	1907 (93.71)	8,364 (93.26)	0.47
Mather’s health	Unhealthy	967 (8.79)	199 (9.78)	768 (8.56)	
	Healthy	10,036 (91.21)	1836 (90.22)	8,200 (91.44)	0.08
Per capita household income	Minimum 25%;	3,250 (29.54)	542 (26.63)	2,708 (30.2)	
	Lower middle 25%;	3,804 (34.57)	657 (32.29)	3,147 (35.09)	
	Middle to upper 25%;	2,690 (24.45)	522 (25.65)	2,168 (24.17)	
	Up to 25%	1,259 (11.44)	314 (15.43)	945 (10.54)	<0.01
Family size	2–7	9,882 (89.81)	1940 (95.33)	7,942 (88.56)	
	8–14	1,121 (10.19)	95 (4.67)	1,026 (11.44)	<0.01
Father’s education	Primary education	3,781 (34.36)	695 (34.15)	3,086 (34.41)	
	Secondary education	6,169 (56.07)	1,113 (54.69)	5,056 (56.38)	
	Higher education	1,053 (9.57)	227 (11.15)	826 (9.21)	0.02
Mather’s education	Primary education	4,562 (41.46)	802 (39.41)	3,760 (41.93)	
	Secondary education	5,570 (50.62)	1,017 (49.98)	4,553 (50.77)	
	Higher education	871 (7.92)	216 (10.61)	655 (7.3)	<0.01
Illness management	Scientific care	6,723 (61.10)	903 (44.37)	5,820 (64.9)	
	Self-medication	4,280 (38.90)	1,132 (55.63)	3,148 (35.1)	<0.01
Number of illness times	The number of illness times in the past month	0.40 (0.81)	0.33 (0.67)	0.42 (0.84)	<0.01
HAZ	Height-for-age *Z*-score	−0.53 (1.95)	−0.38 (1.87)	−0.57 (1.97)	<0.01
BMIZ	BMI-for-age *Z*-score	0.33 (1.68)	0.45 (1.64)	0.31 (1.69)	<0.01
WAZ	Weight-for-age *Z*-score	0.07 (1.33)	0.23 (1.42)	0.04 (1.31)	<0.01

*Frequency and composition ratio (in brackets) or mean and standard deviation (in brackets) are reported.

### 3.2 Child health and urban–rural medical insurance integration

In this study, we analyzed the longitudinal impact of China’s urban–rural medical insurance integration policy on children’s health at two critical time points: 2017 and 2018. Tables 3, 4 present the relationship between the policy and children’s health indicators. Table 3 focuses on the 2017 policy-implementation group. Results show that the policy significantly reduced children’s illness incidence (coefficient: -0.097, 95% CI: −0.193 to −0.001) and improved their BMI – for – age *Z*-scores (coefficient: 0.194, 95% CI: 0.009 to 0.379), indicating better nutrition. However, no significant effects were found on height – for – age *Z*-scores (HAZ) and weight – for – age *Z*-scores (WAZ). Table 4 examines the 2018 policy – implementation group and finds no significant policy impact on any health indicators. The main difference between this and the 2017 policy wave was the policy implementation time. The lack of effect in 2018 may be due to policy implementation factors. The implementation of the policy may face obstacles such as low acceptability, poor feasibility or adaptation problems in the early stage (31). Also, the implementation period was perhaps too short for the policy to show results. Overall, the urban–rural medical insurance integration policy was significantly associated with lower illness risk and higher nutrition levels in children (*p* < 0.05), but had limited impact on other health indicators.

**Table 3:** Estimates of the effect of URRBMI implemented in 2017 on insured children health.

Variables	Number of illness times	BMIZ	HAZ	WAZ
Integration	−0.097**	0.194**	0.058	−0.008
	(−0.193,−0.001)	(0.009,0.379)	(−0.163,0.280)	(−0.195,0.180)
Control variables	YES	YES	YES	YES
Constant	0.482	0.053	−0.511	0.240
	(−0.106,1.070)	(−0.684,0.790)	(−1.468,0.446)	(−0.485,0.965)
R-squared	0.491	0.539	0.611	0.688
Observations	6,540	5,340	5,714	4,044
Individual FE	YES	YES	YES	YES
Year FE	YES	YES	YES	YES

(1) Coefficient and 95% confidence interval (in brackets) are reported; (2) **p < 0.05; ***p < 0.01; (3) Control variables include gender (male; female), age (0–5; 6–10; 11–15), household registration (non-agricultural; agricultural), the quantile of per capita household net income (minimum 25%; lower middle 25%; upper middle 25%; maximum 25%), family size (1–7; 8–14), school attendance (yes; no), caregiver (parents; grandparents; other), whether left behind children (yes; no), birth weight (normal birth weight; low birth weight; Macrosomia), parents’ education (primary education; secondary education; higher education), illness management (scientific care; self-medication) and parental health (unhealthy; healthy).

**Table 4:** Estimates of the effect of URRBMI implemented in 2018 on insured children health.

Variables	Number of illness times	BMIZ	HAZ	WAZ
Integration	0.037	0.117	0.113	−0.077
	(−0.052,0.125)	(−0.097,0.331)	(−0.103,0.329)	(−0.273,0.119)
Control variables	YES	YES	YES	YES
Constant	0.431**	0.587	0.182	0.573
	(0.078,0.783)	(−0.428,1.602)	(−0.818,1.182)	(−0.214,1.360)
*R*-squared	0.452	0.530	0.617	0.606
Observations	5,834	4,653	5,029	3,639
Individual FE	YES	YES	YES	YES
Year FE	YES	YES	YES	YES

(1) Coefficient and 95% confidence interval (in brackets) are reported; (2) *p < 0.10; **p < 0.05; ***p < 0.01; (3) Control variables include gender (male; female), age (0–5; 6–10; 11–15), household registration (non-agricultural; agricultural), the quantile of per capita household net income (minimum 25%; lower middle 25%; upper middle 25%; maximum 25%), family size (1–7; 8–14), school attendance (yes; no), caregiver (parents; grandparents; other), whether left behind children (yes; no), Birth weight (normal birth weight; Low birth weight; Macrosomia), parents’ education (primary education; secondary education; higher education), illness management (scientific care; self-medication) and parental health (unhealthy; healthy).

### 3.3 Robustness test

#### 3.3.1 Parallel trends test

**Figure 1:**
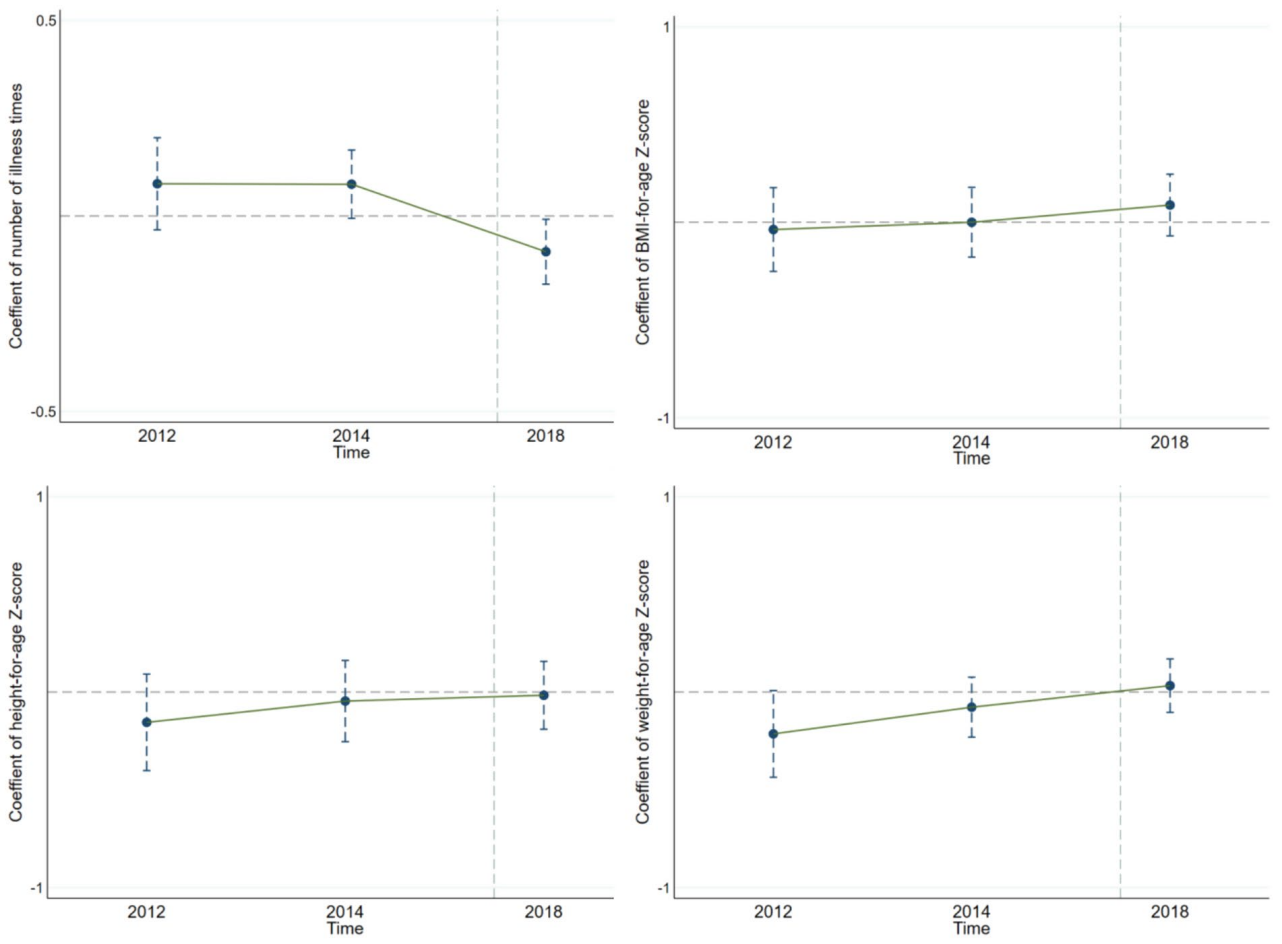
Parallel trend of urban–rural medical insurance integration implemented in 2017.

**Figure 2:**
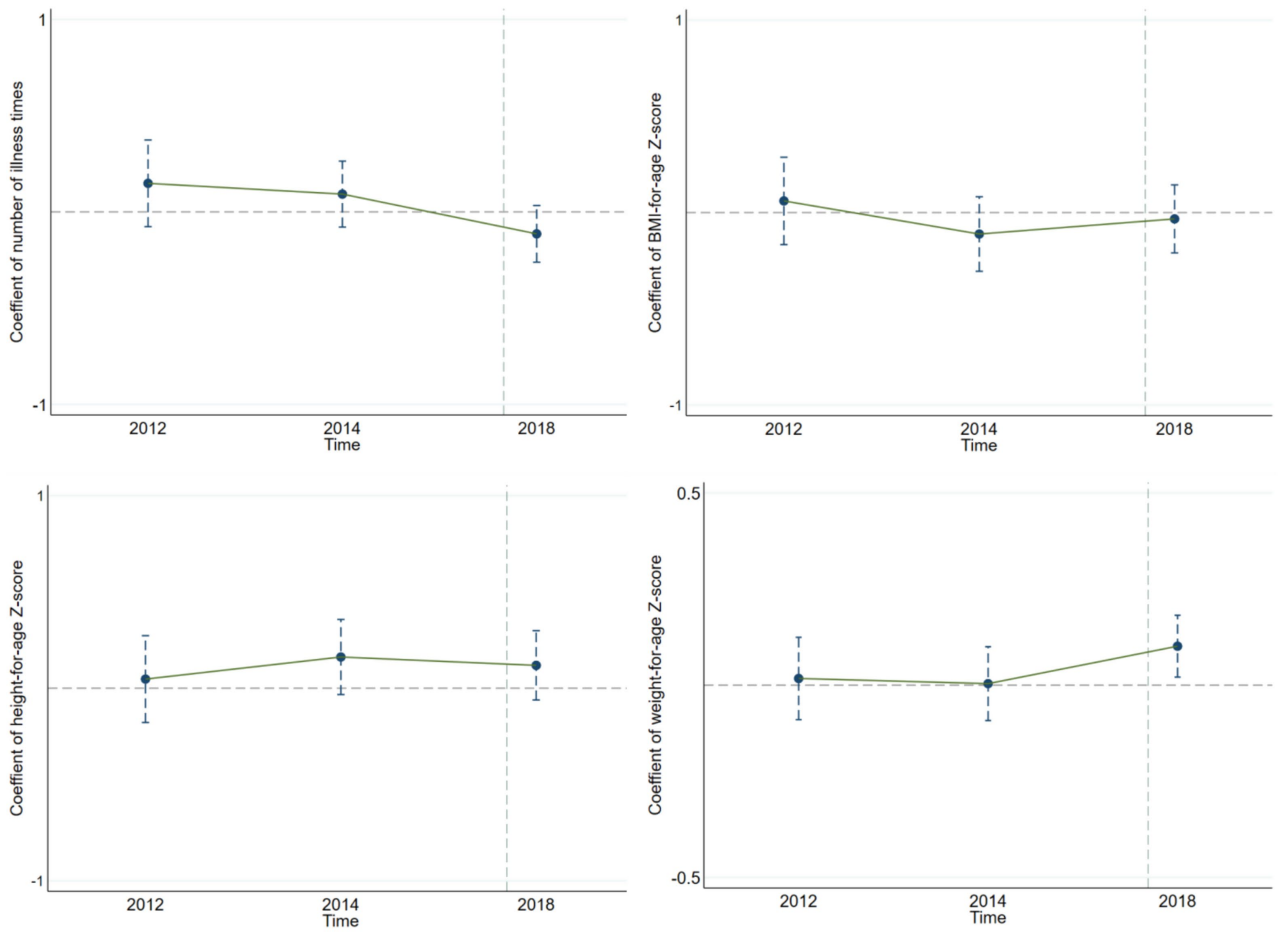
Parallel trend of urban–rural medical insurance integration implemented in 2018.

The parallel trend assumption, a key premise of the DID method, requires that the treatment and control groups have the same development trend before the policy is implemented. Only then can the coefficient of the interaction term between the policy group variable and policy period variable accurately reflect the policy’s independent impact. In this study, we use the event-study method to test the parallel trend assumption. Specifically, the interaction term between the policy group variable and the relative time dummy variable is used as the main explanatory variable, and other control variables are consistent with the benchmark regression model. After the regression analysis, we drew the parallel – trend – test graphs for children’s health indicators (Figures 1, 2). Looking at the results from Figure 1, for the policy implemented in 2017, the interaction-term coefficients before the policy implementation are within the 95% confidence interval that includes 0. This means there were no significant differences between the treatment and control groups before the policy, confirming the parallel trend assumption. Similarly, we drew the parallel-trend graph for the policy implemented in 2018 (Figure 2), and the results also satisfy the parallel trend assumption. This provides a solid basis for our policy-effect assessment.

#### 3.3.2 Placebo tests

**Figure 3:**
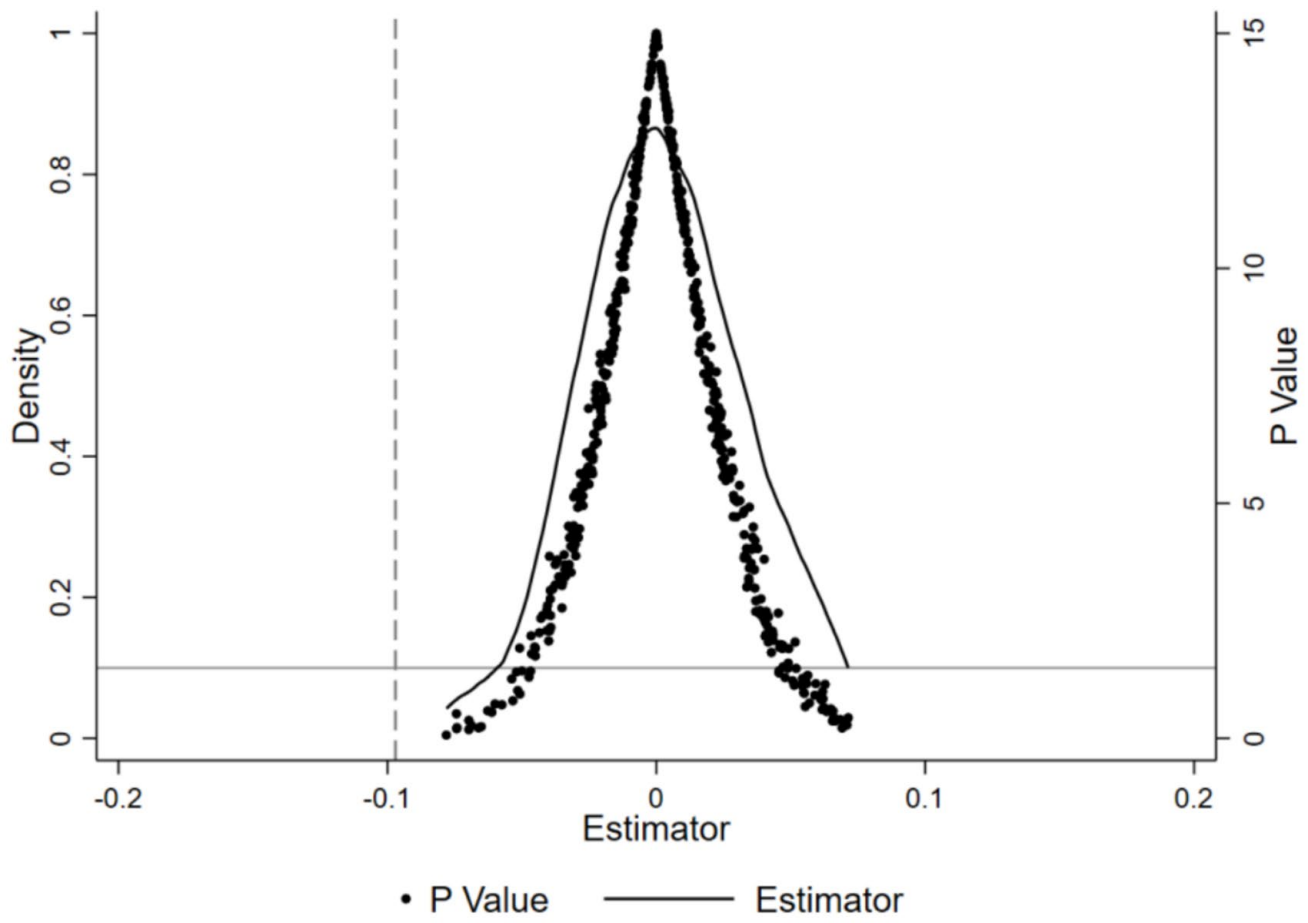
Placebo test with the number of illness times as the outcome variable.

**Figure 4:**
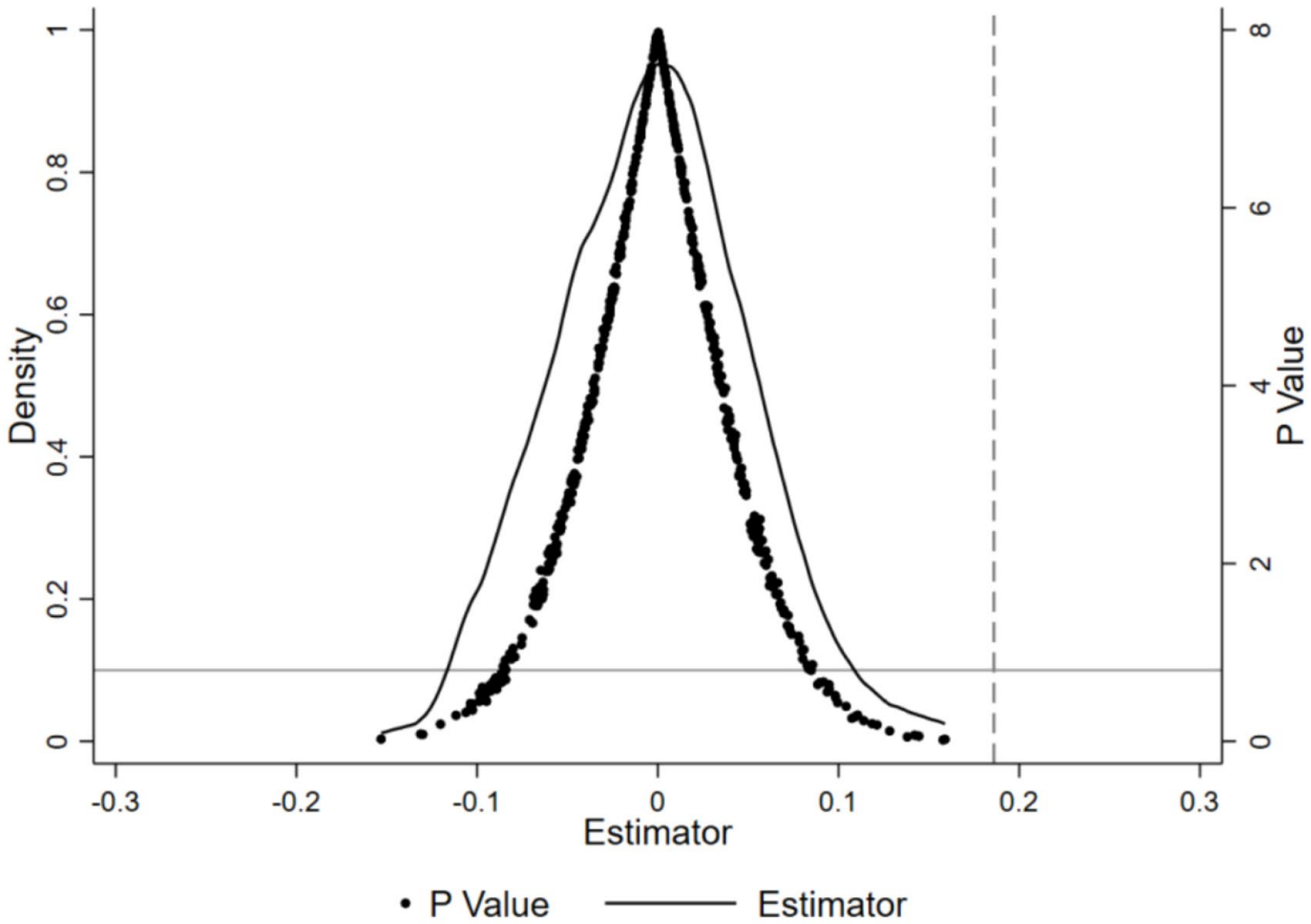
Placebo test with BMI-for age *Z* score as the outcome variable.

To ensure that the effects of rural–urban medical insurance integration on children’s sickness frequency and BMIZ aren’t due to other random factors, a placebo test was used. This test aimed to check if the health improvements from the insurance integration were just by chance. Firstly, 500 random samples were drawn. In each, a “pseudo-interaction term” was randomly assigned to simulate scenarios without the insurance integration’s impact. Then, regression analyses based on model (Equation 1) were done for each sample, giving coefficients and *p*-values. The results of the 500 regressions were aggregated, and their coefficient and *p*-value distributions could be seen in Figures 3, 4. The average coefficients of the “pseudo-interaction term” for children’s sickness frequency and BMIZ were close to 0. This means, without the insurance integration’s effect, these indicators would not change significantly. Also, the coefficient distribution was nearly normal, showing the rationality of the random sampling and result stability. Most *p*-values were above 0.1, so at the 10% significance level, we could not reject the null hypothesis. That is, the “pseudo-interaction term” had no significant effect on children’s sickness frequency and BMIZ. This sharply contrasts with the benchmark regression results, where the insurance integration had significant impacts on children’s sickness and nutrition. So, the positive effects of the insurance integration on children’s sickness frequency and BMIZ are not due to random factors but are reliable and genuine policy outcomes.

### 3.4 Heterogeneity analysis

To more deeply assess the impact of the urban–rural medical insurance integration on children’s health indicators across different groups, we conduct heterogeneity analysis from the dimensions of household registration, family socioeconomic status, and region. This enhances our understanding of the relationship between the integration and children’s health indicators, and offers precise guidance for future interventions. Tables 5–9 present the results of this stratified analysis, revealing significant differences in the policy’s impact on children’s health across groups. Specifically, the 2017 urban–rural medical insurance integration had a marked effect on rural – registered children’s health. As shown in Table 5, the policy’s impact coefficients on the number of illness times and BMIZ for these children were significant at the 10 and 5% levels respectively, aligning with the benchmark regression results. This indicated the integration played a more evident role in improving rural children’s health. Previous studies had revealed the possible reasons behind this outcome. Rural areas, originally suffering from a lack of healthcare resources, have had this gap filled by policies, which has had a more pronounced effect on promoting the health of vulnerable rural children (32). In contrast, urban children, possibly due to information overload and abundant existing resources (33), have shown relatively smaller effects from the policies.

**Table 5:** Estimates of the effect of integration of urban and rural medical insurance on insured children health, grouped by registered residence type.

Variables	Non-agricultural		Agricultural	
	Number of illness times	BAZ	Number of illness times	BAZ
Integration	−0.019	0.078	−0.105*	0.232**
	(−0.233,0.194)	(−0.241,0.397)	(−0.212,0.002)	(0.011,0.454)
Constant	1.800***	−0.974	0.474*	0.287
	(0.808,2.792)	(−2.633,0.686)	(−0.029,0.977)	(−0.450,1.025)
Observations	858	786	5,583	4,475
*R*-squared	0.496	0.661	0.488	0.523
Control variables	YES	YES	YES	YES
Individual FE	YES	YES	YES	YES
Year FE	YES	YES	YES	YES

(1) Coefficient and 95% confidence interval (in brackets) are reported; (2) *p < 0.10; **p < 0.05; ***p < 0.01; (3) Control variables include gender (male; female), age (0–5; 6–10; 11–15), household registration (non-agricultural; agricultural), the quantile of per capita household net income (minimum 25%; lower middle 25%; upper middle 25%; maximum 25%), family size (1–7; 8–14), school attendance (yes; no), caregiver (parents; grandparents; other), whether left behind children (yes; no), Birth weight (normal birth weight; Low birth weight; Macrosomia), parents’ education (primary education; secondary education; higher education), illness management (scientific care; self-medication) and parental health (unhealthy; healthy).

Besides, Table 6 reports the regression results of the impact of the integration of urban and rural medical insurance on children’s health, using the number of illnesses as a health indicator and stratified by socioeconomic status. We found that the results are insignificant. Possible explanations included regional differences in policies and their short implementation span. Table 7 analysis examines the role of family socioeconomic status in influencing the policy’s impacts. The integration positively affected BMIZ in middle-socioeconomic-status children. While insignificant for other groups, the coefficient direction was consistent with the main regression results, suggesting a potential positive effect in different socioeconomic status (SES) groups, particularly pronounced in middle-status children. According to the social resource allocation theory (34), families of medium SES, positioned in the middle tier of social resource acquisition, could swiftly seize and utilize policy benefits, thanks to their information – acquisition ability and social capital. Unlike high – SES families with ample resources and low policy dependence, or low-SES families constrained from fully benefiting, they strike a balance that puts them in a better position to leverage policy advantages. From a regional perspective, Tables 8, 9 show regional disparities in the policy’s effects. In eastern China, the policy significantly reduced children’s illness frequency at the 10% level, while in western China, it significantly improved children’s BMIZ at the same level, indicating better nutrition development. In summary, the urban–rural medical insurance integration’s impact on children’s health varies significantly across household registration types, family socioeconomic status, and regions. These findings deepen our understanding of the policy’s effects and provide empirical support for more targeted child – health interventions.

**Table 6:** Estimated impact of urban–rural medical insurance integration on insured children’s health by SES, with the number of illness times as the outcome variable.

Groups	Lowest SES	Middle SES	Highest SES
Integration	−0.275	−0.129	0.042
	(−0.614,0.065)	(−0.348,0.089)	(−0.188,0.273)
Constant	0.827	0.559	−0.834
	(−0.254,1.907)	(−0.501,1.619)	(−3.742,2.075)
Observations	1,146	1,436	834
*R*-squared	0.557	0.535	0.600
Control variables	YES	YES	YES
Individual FE	YES	YES	YES
Year FE	YES	YES	YES

(1) Coefficient and 95% confidence interval (in brackets) are reported; (2) Control variables include gender (male; female), age (0–5; 6–10; 11–15), household registration (non-agricultural; agricultural), the quantile of per capita household net income (minimum 25%; lower middle 25%; upper middle 25%; maximum 25%), family size (1–7; 8–14), school attendance (yes; no), caregiver (parents; grandparents; other), whether left behind children (yes; no), Birth weight (normal birth weight; Low birth weight; Macrosomia), parents’ education (primary education; secondary education; higher education), illness management (scientific care; self-medication) and parental health (unhealthy; healthy).

**Table 7:** Estimated impact of urban–rural medical insurance integration on insured children’s health by SES, with BMIZ as the outcome variable.

Groups	Lowest SES	Middle SES	Highest SES
Integration	0.115	0.381**	0.350
	(−0.584,0.815)	(0.005,0.756)	(−0.133,0.833)
Constant	0.510	−0.884	0.220
	(−1.472,2.491)	(−2.639,0.871)	(−1.943,2.384)
Observations	203	1,664	1,053
*R*-squared	0.525	0.628	0.616
Control variables	YES	YES	YES
Individual FE	YES	YES	YES
Year FE	YES	YES	YES

(1) Coefficient and 95% confidence interval (in brackets) are reported; (2) **p < 0.05; (3) Control variables include gender (male; female), age (0–5; 6–10; 11–15), household registration (non-agricultural; agricultural), the quantile of per capita household net income (minimum 25%; lower middle 25%; upper middle 25%; maximum 25%), family size (1–7; 8–14), school attendance (yes; no), caregiver (parents; grandparents; other), whether left behind children (yes; no), Birth weight (normal birth weight; Low birth weight; Macrosomia), parents’ education (primary education; secondary education; higher education), illness management (scientific care; self-medication) and parental health (unhealthy; healthy).

**Table 8:** Estimated impact of urban – rural medical insurance integration on insured children’s health by region, with the number of illness times as the outcome variable.

Variables	Eastern region	Central region	Western region
Integration	−0.152*	−0.139	−0.117
	(−0.320,0.016)	(−0.495,0.217)	(−0.274,0.040)
Constant	0.785*	0.317	0.383
	(−0.038,1.608)	(−0.523,1.158)	(−0.881,1.647)
Observations	1,565	1812	1937
*R*-squared	0.475	0.487	0.536
Control variables	YES	YES	YES
Individual FE	YES	YES	YES
Year FE	YES	YES	YES

(1) Coefficient and 95% confidence interval (in brackets) are reported; (2) *p < 0.10; (3) Control variables include gender (male; female), age (0–5; 6–10; 11–15), household registration (non-agricultural; agricultural), the quantile of per capita household net income (minimum 25%; lower middle 25%; upper middle 25%; maximum 25%), family size (1–7; 8–14), school attendance (yes; no), caregiver (parents; grandparents; other), whether left behind children (yes; no), Birth weight (normal birth weight; Low birth weight; Macrosomia), parents’ education (primary education; secondary education; higher education), illness management (scientific care; self-medication) and parental health (unhealthy; healthy).

**Table 9:** Estimated impact of urban – rural medical insurance integration on insured children’s health by region, with BMIZ as the outcome variable.

Groups	Eastern region	Central region	Western region
Integration	−0.023	0.544	0.351*
	(−0.293,0.246)	(−0.289,1.377)	(−0.017,0.718)
Constant	0.025	0.252	−0.328
	(−1.317,1.367)	(−0.813,1.318)	(−1.906,1.249)
Observations	2,193	1842	1914
*R*-squared	0.566	0.539	0.521
Control variables	YES	YES	YES
Individual FE	YES	YES	YES
Year FE	YES	YES	YES

(1) Coefficient and 95% confidence interval (in brackets) are reported; (2) *p < 0.10; (3) Control variables include gender (male; female), age (0–5; 6–10; 11–15), household registration (non-agricultural; agricultural), the quantile of per capita household net income (minimum 25%; lower middle 25%; upper middle 25%; maximum 25%), family size (1–7; 8–14), school attendance (yes; no), caregiver (parents; grandparents; other), whether left behind children (yes; no), Birth weight (normal birth weight; Low birth weight; Macrosomia), parents’ education (primary education; secondary education; higher education), illness management (scientific care; self-medication) and parental health (unhealthy; healthy).

### 3.5 Mechanism test

To further explore the mechanism of the urban–rural medical insurance integration policy on children’s health, we used CMI participation as a moderator. As shown in Table 10, when the number of illness times was the health outcome, the interaction term was −0.195, significantly negative at the 95% confidence level, aligning with the direction of the main effect. This suggests that CMI participation strengthens the urban–rural medical insurance integration policy’s positive impact on children’s health. However, when BMIZ was the health outcome, the interaction term was insignificant, indicating no moderating role of CMI participation.

**Table 10:** Estimates of the moderating effect of participation in commercial medical insurance.

Variables	Number of illness times	BMIZ
Integration	−0.092**	0.198*
	(−0.174,−0.010)	(−0.017,0.413)
CMI	0.024	−0.043
	(−0.047,0.096)	(−0.209,0.123)
Integration * CMI	−0.195**	−0.158
	(−0.389,-0.002)	(−0.447,0.131)
Control variables	YES	YES
Constant	0.432	0.054
	(−0.095,0.959)	(−0.735,0.842)
*R*-squared	0.494	0.563
Observations	6,520	5,317
Individual FE	YES	YES
Year FE	YES	YES

(1) Coefficient and 95% confidence interval (in brackets) are reported; (2) *p < 0.10, **p < 0.05; (3) Control variables include gender (male; female), age (0–5; 6–10; 11–15), household registration (non-agricultural; agricultural), the quantile of per capita household net income (minimum 25%; lower middle 25%; upper middle 25%; maximum 25%), family size (1–7; 8–14), school attendance (yes; no), caregiver (parents; grandparents; other), whether left behind children (yes; no), birth weight (normal birth weight; low birth weight; Macrosomia), parents’ education (primary education; secondary education; higher education), illness management (scientific care; self-medication) and parental health (unhealthy; healthy).

## 4 Discussion

### 4.1 In-depth analysis of research results

To promote equitable access to basic medical security for both urban and rural residents, China’s State Council rolled out a policy to unify urban and rural medical insurance nationwide. This sequential local implementation created a quasi-experimental setting. Our study based on this setting revealed that the 2017 integration policy cut down illness episodes and enhanced child nutrition, particularly for rural–registered and middle – SES children. Rural-registered children, who were previously enrolled in the NRCMS, switched to the URRBMI after integration. Compared to the NRCMS, the URRBMI offers more benefits in terms of overall planning and reimbursement ratio (35, 36). Middle-SES families, with better economic and educational resources, can utilize the insurance more effectively. They can afford medical costs and benefit more from reimbursements, thus improving child nutrition (37). Moreover, the results indicated that in the eastern region, children’s illness episodes are significant at the 10% level. This is likely due to the advanced medical infrastructure, higher-level medical technology, and abundant medical resources in the eastern region, which enable timely and effective treatment for children. In contrast, in the western region, the BMIZ coefficients are significant at the 10% level. Despite having fewer medical resources, the insurance policy probably increased children’s use of medical services, allowing those previously untreated due to financial constraints to receive care and improve their nutrition (38).

In the analysis of the moderating effect, it was found that participation in CMI strengthened the positive impact of the urban–rural medical insurance integration policy on children’s health. URRBMI can cover some medical expenses and reduce the economic burden on families when children fall ill, but its coverage and payment ratio are limited. The supplementary role of CMI (39) not only expands the coverage to include more medicines and treatment methods but also enhances the accessibility of medical services (40). This enables children to obtain high-quality medical services more promptly, thereby reducing the number of times they get sick. In addition, the preventive health care services provided by CMI help detect and intervene in children’s health problems at an early stage. In the long run, this dual-protection mechanism jointly reduces the frequency of children’s illnesses and promotes their health. It is suggested that we continue promoting the coordinated development of commercial and basic medical insurance, and expand the role of CMI in achieving the goal of universal health (41). However, this suggestion should be considered as an exploratory direction for future policy-making, rather than a definitive conclusion directly drawn from our current data.

Based on the economic concept of “policy lag” (42, 43), the lag affecting the policy effect can be divided into implementation lag and impact lag. The impact lag refers to the delay between the time of strategy implementation and the time at which the strategy effect can be observed. In the 2018 wave, there was insufficient evidence on the impact of the integration policy on children’s health. This may be because the policy has a clear transition period. It is a gradual process from the popularization of insurance, public awareness to service utilization (44). In the 2018 wave of children who were surveyed by the CFPS in the year of implementation of the policy, the improvement in health level was not fully apparent. The 2017 wave of children and their parents experienced more than a year of adaptation to the policy, with an increased impact on children’s health levels.

### 4.2 International comparison of the study findings

Ghana’s National Health Insurance Scheme (NHIS) is a government-funded health insurance system aiming to provide basic medical security and reduce the medical burden for all citizens. In terms of promoting child health, it is similar to China’s urban–rural health insurance integration policy, significantly improving the health of insured children. Among children under five, those with health insurance have a lower probability of anemia than those without (45). Compared with babies born before the NHIS was implemented, the infant mortality rate after the NHIS was introduced dropped by 50% (46), and it also increased children’s access to and use of health services (47). This shows that both health insurance policies can positively impact child health. However, due to differences in the medical systems, policy designs, and economic development levels of the two countries, there are differences in fairness protection. For example, the biggest distinction between the NHIS and the urban–rural health insurance integration policy is the coverage. The NHIS covers all residents of Ghana, regardless of urban or rural areas, formal or informal sector workers. But except for specific groups like children and the older adult who are exempt from paying for participation, most Ghanaians join the NHIS on a voluntary basis. This leads to the universal insurance coverage not being fully realized (48), with the poor and those with low education levels being left out (49, 50). Moreover, in terms of protection benefits, the NHIS does not sufficiently protect the property risks of insured children’s families. Families still have to pay out-of-pocket fees for consultations and medicines (51), and they cannot avoid catastrophic health expenditures (CHE) (52).

Indonesia’s universal health insurance (JKN) program is formed by merging other social security insurance programs with the government’s insurance plan. After unifying the health insurance fund, it becomes a single-payer system covering all citizens (53). This is similar to the direction of the urban–rural health insurance integration policy. In terms of promoting child health, the JKN reduces stunting among children under five in urban poor communities in Indonesia (54) and significantly cuts the out-of-pocket medical expenses of insured children’s families (55), greatly improving maternal health care service quality (56). Compared with China’s urban–rural health insurance integration policy, both aim to promote child health and have similar effects, but their implementation backgrounds and specific policy measures differ. China’s policy focuses on integrating urban-resident insurance and the New Cooperative Medical Scheme to enhance fairness, while Indonesia’s JKN emphasizes universal coverage and health-service accessibility (57). This difference likely stems from the different social-cultural backgrounds and stages of development of the health-security systems in the two countries.

It is worth noting that while these international comparisons provide valuable insights, they are based on the specific contexts of each country. The application of these comparisons to other low- and middle-income countries should be done with caution, considering the unique circumstances of each nation. Our study introduces two cases of health insurance policies from developing economies: Ghana’s NHIS and Indonesia’s JKN. Both cover all citizens nationwide. China’s urban–rural resident basic medical insurance system, formed by integrating urban-resident insurance and the New Cooperative Medical Scheme, mainly targets non-employed and informal-sector residents in urban and rural areas. This offers experience for further integrating urban employee basic medical insurance in the future to achieve a single insurance project covering everyone. Meanwhile, the fairness-related issues in Ghana’s NHIS, conflicting with the goal of universal health coverage, provide valuable lessons for China’s subsequent health-care system reform. It prompts China to stress achieving health equity more in health-care reforms, focusing on health-service accessibility, quality, and sustainability to ensure universal health coverage is realized not only formally but also in substantive content. However, these suggestions are based on a broad interpretation of the findings and should be viewed as potential directions for further exploration rather than definitive policy recommendations.

### 4.3 The contradiction or consistency with the previous reform in China

In China’s past medical insurance reforms, insurance coverage and hospitalization expense reimbursement rates have risen significantly, boosting medical service usage and enhancing fairness in services across and within regions (58). The NCMS has bolstered rural residents’ medical security and improved rural children’s health (21, 22). The urban–rural health insurance integration policy aligns with these reforms in goals, aiming to refine the medical security system, narrow urban–rural gaps, and promote health equity. It has also achieved positive results in improving rural children’s health, continuing and deepening previous reforms. For instance, the NCMS offered basic medical security to rural residents, and the integration policy has further optimized resource allocation and raised the protection level, enabling rural children to access better medical services. However, compared with past reforms, the urban–rural health insurance integration policy may encounter new challenges in implementation. The integration of different regional medical insurance systems disrupts the urban–rural dual-track system’s restrictions. In practice, it is constrained by the established interest patterns and institutional inertia of the dual-track system, conflicting with the stable operation under the previous single medical insurance system. Moreover, the integration policy demands a higher level of medical security than previous single-system reforms. Yet, current urban–rural medical insurance policies still need strengthening in protection levels and sustainable development (59, 60). In summary, the integration policy’s positive impact on child health matches the goals of past reform efforts. The existence of these contradictions or differences reflects the need to address diverse issues and challenges at different stages of medical insurance reform, requiring careful balancing and optimization in policy design and implementation. These conclusions are drawn from the current data and analysis, but further research is needed to fully understand the long-term implications and broader applicability of these findings.

### 4.4 Significance and limitations of this study

This study evaluates the effect of China’s rural–urban medical insurance integration on children’s health through empirical analysis. This not only verifies previous research conclusions but also offers evidence for policy refinement and new reform directions. We compare the health effects of the policy on children from different family socioeconomic backgrounds, regions, and household registration types. This helps the government and society better implement medical insurance reforms, focus on vulnerable groups, and reduce health disparities among different groups. This contributes to social equity, harmony, and sustainable development. In the long run, children’s health status impacts their adult education, labor productivity, and quality of life, which in turn affects their ability to contribute to society. Thus, this research highlights the social returns of investing in children’s health, encouraging continuous attention and investment in this area. Globally, this study offers references for other countries’ children’s health policies and promotes international cooperation in this field. By comparing with international policies and research, it provides empirical support for global health policy optimization, reduces health inequality, and advances the UN’s health-related Sustainable Development Goals. The social returns of children’s health investment revealed in this study can inform the development of more scientific and reasonable global children’s health policies.

This study has several limitations that should be acknowledged. The DID approach relies on the parallel trends assumption, which we cannot fully confirm due to the observational nature of the data and potential unobserved confounding factors. Although we controlled for observed variables, unmeasured confounding variables or selection bias may still exist, such as the quality of the family living environment and medical services. The absence of physiological function indicators for children’s health restricts our ability to comprehensively assess the policy’s impact. Additionally, by the beginning of 2020, the integration of urban and rural medical insurance had been largely implemented nationwide, leaving no corresponding control group in the survey year of 2020. This limits our ability to examine the long-term effects of the policy on children’s health. In future research, additional robustness and falsification tests could be conducted to address potential endogeneity issues. Collecting more comprehensive data on children’s physiological health indicators and extending the study period would also provide a more accurate assessment of the policy’s long-term effects.

## 5 Conclusion

This study set out to explore the impact of integrating urban and rural medical insurance systems on the health status of children in China, a critical issue given the persistent disparities in healthcare access and child well-being across regions and socioeconomic groups. With healthcare reform being central to China’s broader social policy goals, understanding how institutional changes in insurance coverage affect vulnerable populations, particularly children, is of both academic and policy significance. Using nationally representative longitudinal data and robust quasi-experimental methods, the study provides evidence on how this integration policy has influenced children’s physical health and nutritional outcomes, with additional attention to the role of commercial insurance and household-level heterogeneity.

The integration of urban and rural medical insurance has a positive impact on children’s health. The integration of urban and rural medical insurance has promoted the improvement of children’s physical fitness and nutritional status. Participation in commercial medical insurance enhances the positive impact of the integration of urban and rural medical insurance on children’s physical fitness. However, the policy has a lag, and the initial stage of the policy needs the government to control and optimization, and the insured people’s attention and active adaptation. The integration policy of urban and rural medical insurance is heterogeneous and has a greater impact on rural household registration and middle-SES children.

To further enhance the impact and utility of the conclusion, based on the findings of this study, we propose specific policy suggestions. To address the policy implementation lag, governments at all levels should strengthen the monitoring and evaluation mechanisms of the policy implementation process, establish real-time feedback channels to promptly identify and solve problems. At the same time, enhance the training and publicity of the policy to improve the understanding and operational capacity of the implementers. Also, strengthen the regulation of commercial medical insurance to promote its synergy with basic medical insurance. In response to the heterogeneous effects of the policy, it is necessary to formulate differentiated policy measures. For example, for rural household registration children, increase the investment in rural medical resources, improve the service level of rural medical institutions, and reduce the medical burden of rural families through measures such as medical subsidy policies. For children from low-and middle-income families, adjust the medical insurance reimbursement standards and benefit packages appropriately according to their actual needs to improve the fairness and effectiveness of the medical insurance system. In conclusion, this study could offer valuable insights for policy makers in constructing and developing China’s children’s medical insurance system and informative references for other LMICs in their children’s health policy-making.

## Data Availability

Publicly available datasets were analyzed in this study. This data can be found: https://www.isss.pku.edu.cn/cfps/index.htm.
